# Avian Metapneumovirus: Current Knowledge, Critical Gaps, and Future Directions in Transmission, Pathogenesis, and Control Across Poultry Systems

**DOI:** 10.3390/v18070781

**Published:** 2026-07-16

**Authors:** Abhijith Anil, Biswash Ghimire, Menuka Bhandari, Kush Kumar Yadav

**Affiliations:** 1 Department of Animal Sciences, College of Food, Agricultural and Environmental Sciences, The Ohio State University, Wooster, OH 44691, USA; anil.19@buckeyemail.osu.edu (A.A.); ghimire.73@buckeyemail.osu.edu (B.G.); 2 Research and Analytical Service Core, College of Food, Agricultural and Environmental Sciences, The Ohio State University, Wooster, OH 44691, USA; bhandari.72@osu.edu

**Keywords:** avian, metapneumovirus, turkey, production, sustainability, animal, health

## Abstract

Avian metapneumovirus (aMPV) is an economically significant respiratory virus of poultry that causes major losses due to reduced egg production and increased susceptibility to secondary infections. It has global significance in poultry production systems and mainly affects turkeys, chickens, and ducks. It was first detected in South Africa in 1978 and is classified into four distinct subtypes (A–D) based upon sequence divergence of the glycoprotein (G) gene. Subtype C has historically been the dominant subtype in the US, first detected in the late 1990s, causing approximately $15 million economic loss per year in Minnesota alone. However, after a decade, aMPV re-emerged in the fall of 2023. Subtype A in California and B in North Carolina was detected for the very first time in the US, impacting multiple states, with an estimated $112 million loss in Minnesota alone. The most recent US Animal Health Association (USAHA) annual survey has now ranked aMPV as the most frequently reported issue in turkey production. Even after four decades of virus identification, areas such as transmission routes, host–virus interactions and tissue tropism within the host are not studied in detail. Addressing these gaps via current modernized research tools is crucial in minimizing the impact of aMPV on the poultry industry. Therefore, this review article summarizes current knowledge on aMPV while identifying critical research gaps and emphasizing future research directions for combating aMPV and supporting long-term sustainability of the turkey industry.

## 1. Introduction

Avian metapneumovirus (aMPV) is a significant respiratory infectious agent of birds with a wide geographical distribution and substantial economic impacts. It primarily affects the respiratory and reproductive tracts of turkeys, chickens and ducks, leading to severe respiratory distress, nasal discharge, poor eggshell quality, and a drop in egg production [1,2,3]. The virus causes turkey rhinotracheitis (TRT) in turkeys and swollen head syndrome (SHS) in chickens, terms that reflect the characteristic clinical signs and lesions observed in each species [4]. Interestingly, aMPV has been detected in almost all poultry-producing countries except Australia, likely due to the country’s strict biosecurity measures and geographical isolation [4,5]. This highlights the extensive global distribution of aMPV that demands the detailed analysis of its transmission biology and pathogenesis.

**Figure 1:**
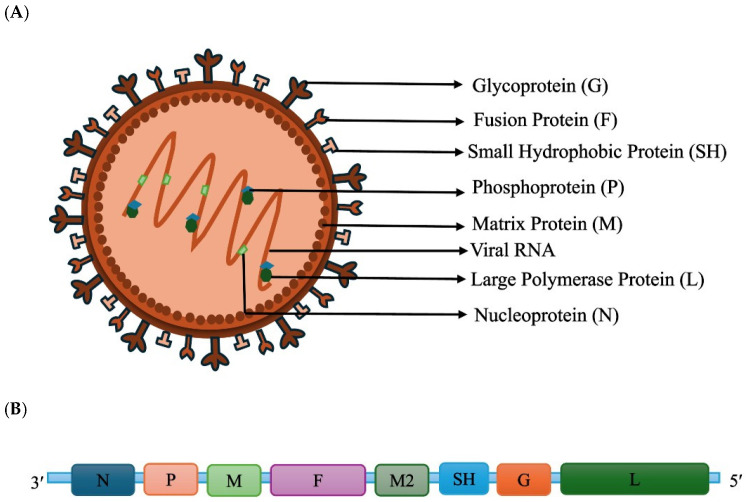
Schematic demonstration of aMPV. (**A**) aMPV viral structure. (**B**) Genomic organization of aMPV. N: nucleoprotein, P: phosphoprotein, M: matrix protein, F: fusion protein, M2: second matrix protein, SH: small hydrophobic protein, G: glycoprotein, L: large polymerase protein.

aMPV was initially reported in 1978 in turkeys in South Africa and was subsequently detected across multiple continents [6]. In the US, the virus was first documented in 1997 [4,7,8]. aMPV is classified under order *Mononegavirales*, family *Pneumoviridae* and genus *Metapneumovirus*. Metapneumovirus species are characterized by the absence of the NS1 and NS2 genes, which encode non-structural proteins and are defining features of other members of the genus *Orthopneumovirus* within the same family. The genus *Metapneumovirus* comprises human metapneumovirus (hMPV) and aMPV [9,10]. aMPV viral particles show pleomorphic, fringed, generally spherical forms measuring 80–200 nm in diameter and filamentous forms with surface projections measuring 80–100 nm in diameter (Figure 1A) [4,11]. The viral RNA is single-stranded, non-segmented, and negative-sense, with a genome length of approximately 13.3–14 kb [9,10]. aMPV has a genome consisting of eight genes that encode the fusion protein (F), phosphoprotein (P), matrix protein (M), nucleoprotein (N), second matrix protein (M2), attachment glycoprotein (G), small hydrophobic protein (SH), and large polymerase protein (L) (Figure 1B) [4,10]. Among the aMPV genes, the G gene, which encodes the attachment glycoprotein, is the most genetically heterogeneous gene in the genome, showing extensive sequence variability. Based on the sequence divergence of the G gene, aMPV is classified into four subtypes: A, B, C and D [12,13,14].

Subtype A and B are widely distributed globally, whereas in the US, subtype C was historically the most prevalent aMPV strain. The country remained free of aMPV for more than ten years, largely due to strict biosecurity practices and the use of live attenuated vaccines [3,8]. However, in 2023, subtypes A and B were identified in the US and caused disease outbreaks in commercial poultry flocks, marking their first detection in the country [3,8]. These outbreaks have created substantial economic losses in the poultry industry nationwide. A study by the Minnesota Turkey Growers Association reported that the turkey industry in Minnesota experienced approximately $112 million in lost sales in 2024 due to aMPV [15]. Although mortality is typically low, it may rise substantially when secondary bacterial infections occur, such as *Mycoplasma gallisepticum*, *Escherichia coli* and *Ornithobacterium rhinotracheale* [4,16,17]. Transmission occurs primarily through direct contact with infected birds, particularly via infectious aerosols and respiratory exudates [18,19]. Indirect transmission is also possible through contaminated equipment, litter, personnel and vehicles, all of which can serve as important sources of viral spread [4].

Diagnosing aMPV based on clinical and pathological findings is challenging, as its clinical features are generalized and closely resemble those of other respiratory diseases such as Newcastle disease and avian influenza. Therefore, molecular tools such as reverse transcription polymerase chain reaction (RT-PCR) and sequencing are crucial for reliable identification and characterization of the virus [4,14]. At present, there is no targeted therapeutic intervention for aMPV. Preventative and control strategies depend on strict biosecurity practices and vaccination programs. The US currently lacks USDA-approved vaccines for aMPV subtypes A and B, forcing reliance on European imports and highlighting the importance of developing domestic vaccine options [2]. The 2024–2025 outbreak in the US demanded the reliance on European vaccine manufacturers like Vaxxinova, Boehringer-Ingelheim, and HIPRA, which supply live attenuated or killed vaccines primarily derived from European aMPV-A and B strains. This reliance further contributes to the economic impact due to the costs associated with vaccine purchase and importation.

In this review, we discuss the current epidemiology, global distribution, viral evolution, host range, transmission routes, environmental persistence, pathogenesis, immune modulation, diagnostics, and surveillance of aMPV. We also explore available vaccines, industry-level impacts, existing knowledge gaps and future research directions for combating aMPV and supporting long-term sustainability of the turkey industry.

## 2. Epidemiology and Global Distribution

The first documentation of aMPV occurred in South Africa in the late 1970s, and the virus has since been detected in almost every country worldwide (Table 1). Subtypes A (aMPV-A) and B (aMPV-B) are the predominant aMPV subtypes reported across Europe, South America, Africa and Asia. Subtype C (aMPV-C) has been documented in the US, Canada, France and multiple countries across Asia [2,14]. The absence of standardized epidemiological tools and structured diagnostic field studies limits the ability to accurately assess aMPV global distribution and its impact on overall poultry production [20,21].

### 2.1. Europe

Turkey rhinotracheitis had been reported in France since 1981, yet none of the early viral isolates appeared to represent the causative agent. In 1985, an outbreak on a well-managed farm in France facilitated successful virus isolation, and the agent was subsequently confirmed to be aMPV. During the same period, the disease had also been identified in the United Kingdom [32,61,62]. The virus was later detected in several other European regions. The early aMPV isolates from Europe included the CVL14/1 strains from the United Kingdom, the 1556 strain from France, the 657/4 strain from Hungary, the 872S strain from Spain, and the 2119 strain from Italy [13,31]. Centered on nucleotide and amino acid sequence comparisons of the G gene, the early isolates from the United Kingdom were classified as subtype A, whereas those from France, Italy, Hungary, and Spain were classified as subtype B [8,13,28,63].

Field serological investigations conducted in Italy have demonstrated the presence of aMPV-A and B in both turkey and broiler farms [20,27]. In Northern Italy, field studies conducted in turkey and broiler farms between 2011 and 2013 indicated that subtype B was the most prevalent strain [2]. Screening for aMPV in broilers, layers, and backyard flocks led to the first molecular identification of the virus in Greece in 2016, which was classified as subtype B [21]. In Belgium, both aMPV-A and aMPV-B were isolated from turkey farms in 1998 [49]. aMPV-B was reported for the first time in a broiler flock with respiratory signs in Romania in 2016, showing a close relationship to Italian field strains of aMPV [59]. In Russia, aMPV subtypes A and B have been identified in chickens and turkeys [20]. During the period of 1987 to 1988, aMPV was isolated in Germany and identified as subtype A in turkeys and subtype B in broiler breeders [34]. In the United Kingdom, early aMPV isolates from turkeys during the late 1980s were mainly subtype A, whereas by the mid-1990s subtype B had become predominant [33]. Current epidemiological studies from Europe highlight aMPV-B as the dominant subtype [3,20,28].

aMPV-C is classified into two lineages: Eurasian aMPV and North American strains. The presence of Eurasian aMPV-C strains was detected in Muscovy ducks in France, confirming the circulation of this virus in the country [26,64]. Between 2017 and 2019, an aMPV subtype C-specific RT-qPCR assay was used to screen wild mallards in the Netherlands, which led to the identification of aMPV-C of Eurasian lineage [65]. Similarly, a subgroup-specific indirect ELISA conducted on blood samples from mallard flocks in Northern Italy showed 100% seroconversion to aMPV subtype C, marking the first report of this finding [29]. The Eurasian lineage of aMPV-C appears to exhibit greater tropism for anatids, as most detected cases have occurred in these species [66]. Two aMPV isolates recovered from turkey flocks in France in 1985 showed no similarity to subtypes A, B, or C and were classified as subtype D, which has not been reported after the initial report [23,63].

### 2.2. North America

The US was initially considered free of aMPV. However, a 1996 respiratory outbreak in Colorado turkeys led to testing in the United Kingdom, where ELISA results first suggested similarity to subtype A. The official isolation of aMPV from the Colorado outbreak was achieved in February 1997 by the National Veterinary Services Laboratories. The combined evidence from matrix and fusion protein phylogeny, along with the isolate’s distinct serological profile compared with European strains, confirmed it to be subtype C [7,67,68]. In March 1997, a similar illness was observed in Minnesota, and aMPV-C was detected in turkeys in the state for the first time using cell culture methods and RT-PCR (Table 2) [46]. Although the initial outbreak occurred in Colorado, the greatest economic impact from aMPV was observed in Minnesota, which ranks first for turkey production in the US [4]. Following its emergence in Minnesota, aMPV was subsequently detected in domestic turkeys in central North American states such as Iowa, Wisconsin, North Dakota and South Dakota using RT-PCR or ELISA [45]. Serum samples obtained from wild birds in Ohio, South Carolina, Georgia and Arkansas showed an 18% seroconversion rate to aMPV antibodies, which on sequencing confirmed as subtype C, underscoring the important role wild birds play in the virus’s epidemiology [69].

Although subtype C historically predominated in US poultry, recent detections in late 2023 and early 2024 indicate that subtypes A and B have entered commercial flocks, with subtype A more common in western states and subtype B more common in eastern states [3,8,47,72]. Evidence suggests that US aMPV-B strains derived from Eastern Asian lineages are connected to European variants, while aMPV-A introductions seem to trace back to Mexico, with genetic links to Asian strains [66]. Interestingly, the route of introduction of these subtypes to the US is still unknown. The National Centre for Foreign Animal Disease (NCFAD) reported Canada’s first detection of aMPV in May 2024, identifying both aMPV-A and aMPV-B in domestic birds [2]. A longitudinal study in breeder hens and pullets from highly productive poultry regions in Mexico identified aMPV-A, confirming its circulation in the country [54].

### 2.3. South America

RT-PCR testing and G-gene sequence analysis of vaccinated and non-vaccinated broiler, breeder, and turkey farms in Brazil revealed the presence of both aMPV-A and aMPV-B, demonstrating the cocirculation of these subtypes in Brazilian commercial flocks [44]. The presence of aMPV was demonstrated in turkey and chicken flocks in Chile in 1997 through ELISA [48]. In Colombia, G-gene targeted RT-PCR detected aMPV-B from laying hens, broilers, breeders, and wild birds [73].

### 2.4. Asia

Subtypes A, B and C were identified in commercial chickens with variable respiratory signs in China, highlighting the presence of the virus in the country [51,52,74]. A novel aMPV-C variant from Sheldrake ducks exhibiting hydrosalpinx fluid syndrome (HFS) was recently identified in China [50]. The presence of aMPV in Japan has been recognized since 1989, with aMPV antibodies detected in chickens exhibiting swollen head syndrome. Both subtypes A and B have been detected in Japan [36]. In Thailand, the first molecular detection of aMPV-B in turkeys was reported in 2022 [75]. Investigations in Malaysia and Vietnam have identified subtype B as the most prevalent aMPV strain [40,76]. Serological analysis of samples using commercial ELISAs capable of detecting aMPV-A, B, and C antibodies in both broiler breeder and layer flocks in India identified seropositive farms, indicating the need for further surveillance of the virus in the country [57,58].

In Turkey, aMPV-B was detected by RT-PCR in broiler flocks sampled between 2017 and 2018, and another study similarly identified aMPV-B in turkeys [55,56]. Serum samples collected from chickens of various age groups in Saudi Arabia between 2007 and 2008 showed a 50% seroconversion rate to aMPV antibodies in birds aged 11–18 weeks [77]. Antibodies to aMPV were also detected in backyard chickens in Saudi Arabia in 2019 [78]. In Jordan, aMPV-B was detected in broilers, layers, and breeder chickens through RT-PCR and ELISA [53]. In Israel, aMPV subtypes A and B were identified in vaccinated chicken as well as in turkey flocks, highlighting the need for more effective vaccination measures [79]. Studies from Iran indicate that subtype B is the most prevalent aMPV strain in chickens and turkeys [80,81].

### 2.5. Africa

The spread of aMPV in most African nations remains poorly characterized, pointing to how much more systematic surveillance is still needed. Initially, the prevalent aMPV strain in Egypt was subtype B, detected in turkeys, but in 2013, subtype A was also identified in turkeys [2,82]. RT-PCR testing in Moroccan broiler flocks identified circulation of aMPV-A and B, with subtype B emerging as the dominant strain [60]. Commercial chicken flocks in Nigeria tested positive for aMPV-B using RT-PCR, with Sanger sequencing validating the subtype [83].

## 3. Phylogenetic Analysis of Avian Metapneumovirus

### Comparison of Virus Clustering Based on Whole-Genome and Full-Length G Sequence

**Figure 2:**
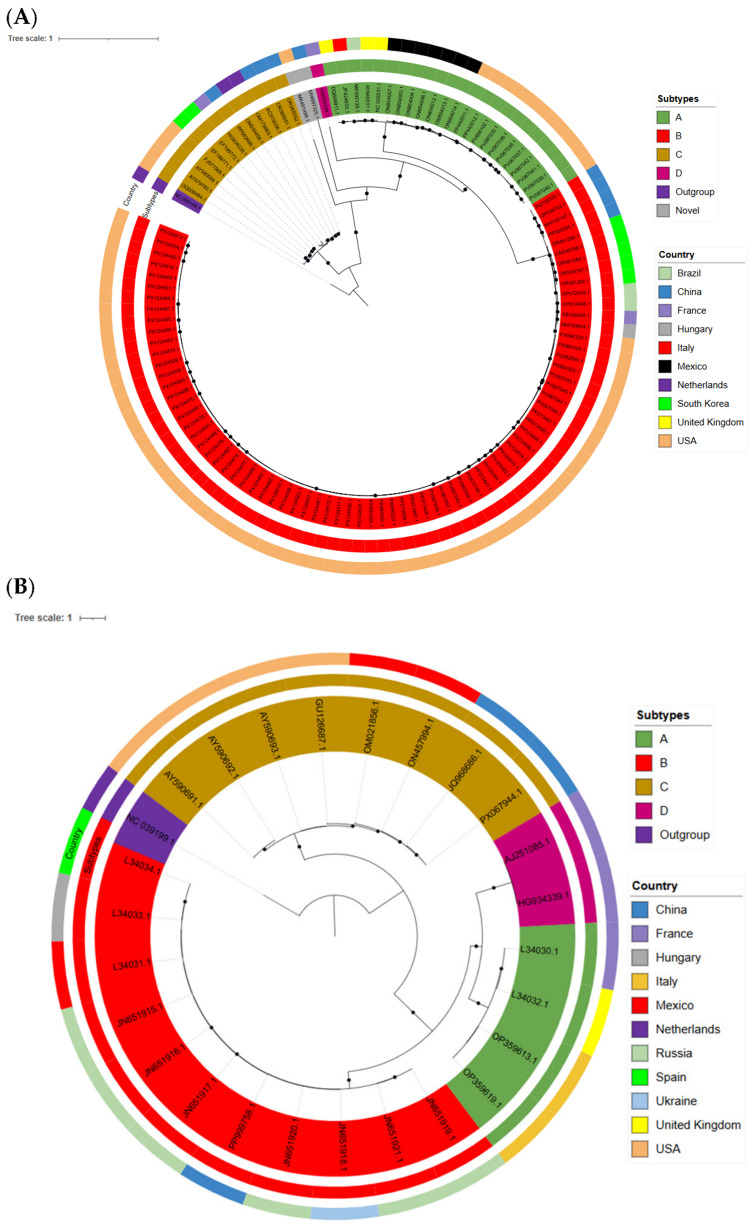
Phylogenetic tree of the whole-genome sequence (n = 117) (**A**) and the full-length glycoprotein sequence (n = 25) (**B**) of aMPV. The rings of the phylogenetic trees indicate the subtypes and country of origin. The tips of the tree are color-coded based on the subtype. To create the phylogenetic tree, both the complete and partial aMPV sequences were obtained from the NCBI GenBank database using the Entrez Direct (search) command-line utility, with the query “[avian metapneumovirus] [Organism]” to filter sequences corresponding to the aMPV. In total, 1146 complete and partial sequences of aMPV were downloaded from NCBI on 08-06-2026. Two phylogenetic trees were constructed using 142 different sequences: whole-genome aMPV sequences (n = 117), and full-length glycoprotein (G) sequences (n = 25). For both trees, human metapneumovirus (**NC_039199**) was included as an outgroup. The multiple sequence alignment of nucleotide sequences was performed in MAFFT v7.110 [85], and the maximum likelihood phylogenetic tree was composed using IQ-TREE v3.0.1 [86]. The tree was constructed using models with 1000 bootstraps (only bootstrap values more than 70% are shown on the tree), and visualized in Interactive Tree of Life [87]. The tree was re-rooted using human metapneumovirus as outgroups. To understand the evolutionary relationships and global subtype distribution of aMPV, phylogenetic analyses were performed using publicly available whole-genome sequences and full-length G-gene sequences. Maximum likelihood phylogenies were inferred in IQ-TREE. For the whole-genome dataset, the GTR + F + I + R3 substitution model was selected as the best-fit model according to the Bayesian Information Criterion (BIC), and branch support was assessed using 1000 ultrafast bootstrap replicates.

Phylogenetic analysis of 117 complete aMPV genomes, and human metapneumovirus as outgroup, demonstrated clear clustering based on the subtypes A, B, C, and D (Figure 2A). The phylogenetic tree further showed that the two major lineages, subtypes A and B, clustered together, whereas subtypes C and D formed separate sister lineages consistent with previous findings [84]. Among the sequences analyzed, subtype B was the most frequently detected subtype isolated predominantly from the USA, whereas subtype D was less frequently isolated. Furthermore, a second phylogenetic analysis was performed using 25 full-length G-gene sequences of aMPV (Figure 2B). For this tree, the K3Pu + F + R2 substitution model was chosen as the best-fit model in reference to BIC. Consistent with the whole-genome analysis, the G-gene phylogeny tree showed subtype-specific clustering and supported the overall evolutionary relationships observed in the complete genome analysis.

## 4. Viral Evolution and Host Range

### 4.1. Worldwide Evolutionary Patterns and Genetic Diversification of aMPV

The emergence and worldwide dissemination of aMPV subtypes illustrate the virus’s evolutionary expansion, shaped by mutation, vaccine-driven selection and host-specific pressures. The genome of aMPV is highly conserved, with stable polymerase and nucleocapsid functions, but the G gene shows extensive diversity and is the major contributor to evolutionary differences among strains [5]. Studies have shown that the time to the most recent common ancestor (TMRCA) for aMPV is estimated to be approximately 200 years ago. Further evolutionary analysis using the Bayesian serial coalescent approach in the Bayesian evolutionary analysis by sampling trees (BEAST) program (incorporates sampling dates and genetic data to estimate evolutionary rates and divergence times) estimated substitution rates for the F and N genes of aMPV-C at 9.3 × 10^−4^ and 1.1 × 10^−3^ substitutions per site per year, respectively [88]. In a similar study, evolutionary rates were estimated for the N, P, F, and M2 genes of aMPV-C. These rates ranged from 1.3 × 10^−3^ to 7 × 10^−3^ substitutions per site per year, indicating the fast-evolving nature of aMPV-C [89]. These estimates underscore how quickly RNA viruses can evolve, driving short generation times, high genetic diversity and the continual emergence of new subtypes. In the same study, phylogenetic analysis of the above genes revealed two distinct aMPV-C lineages, designated C1 and C2. The TMRCA at which these two lineages coalesce is estimated to be between 9 and 14 years [89]. Overall, this evidence points to aMPV-C as a virus that continues to evolve, with its rapid mutation rates and ongoing lineage formation helping drive its continued spread.

For in-depth analysis of the evolutionary dynamics of aMPV-B, researchers analyzed G-gene sequences collected between 1985 and 2019 from nine European countries: Greece, Romania, France, Russia, Italy, Spain, Ukraine, the Netherlands and the United Kingdom. The TMRCA estimates indicated that the first introduction of aMPV-B in Europe occurred in 1981 in France, followed by its introduction into Italy in 1984, from where it subsequently spread to other European countries. The evolutionary rate of 1.21 × 10^−3^ substitutions per site per year falls well within the range typical of RNA viruses, reflecting their high propensity to mutate [28,90]. In Italy, the detection of aMPV-B in vaccinated flocks showed that the circulating field strains were genetically unrelated to both the 1987 Italian isolate and the commonly used vaccine strain [91]. Complete nucleotide and amino acid sequences of the field strain were analyzed and showed the greatest variation in the SH and G proteins. The G mutations were non-synonymous, leading to modifications in viral characteristics as a response to selection pressure. A subtype B virus carrying these changes allowed the virus to infect birds and induce disease even after successful vaccination with an established subtype B vaccine. These changes in the G and SH proteins may have arisen from long-term vaccine-driven selection pressure, leading to the appearance of mutant strains [91]. In the US, the vaccination schedule for aMPV-A and B was generally not followed due to the historical absence of these viruses in the country, which may also have been a predisposing factor for the recent outbreak. Comparative analysis of aMPV-B isolates in Northern Italy from 1987 to 2007 and those circulating in Western Europe before 1994, using the F and G genes, identified clear genetic distinctions. Fusion protein gene sequences showed marked conservation, whereas G protein gene sequences exhibited more variation. The presence of a serine residue at position 382 in the G protein appeared to be unique to the Italian aMPV-B isolates. These changes in the Italian viruses also suggest that the mutations may have arisen from immune pressure, either due to vaccination or exposure to an undetected environmental field strain [92].

In a study conducted at the end of 2024, comparative phylogenomics of samples collected from aMPV-A prevalent areas in the US, aimed at understanding the evolution of circulating aMPV-A, revealed that these strains shared 99.5% identity with US strains in GenBank at both the whole-genome level (WGS) and across all eight genes, including the G gene [93]. The amino acid alignment of the entire G protein also revealed only a few substitutions, suggesting that the currently circulating aMPV-A strain has undergone limited evolution across turkey and chicken. Similarly, for subtype B, whole-genome and G-gene analysis showed that the current isolates were genetically distinct from previously reported strains worldwide but exhibit very high sequence identity among the US aMPV-B strains [93]. The amino acid analysis of aMPV-A and aMPV-B showed only minimal substitutions with the US strains, reflecting their high sequence identity. This high sequence identity in both subtypes potentially indicates limited vaccine-driven selection pressure and a recent introduction of the virus into the country. However, the number of amino acid mutations increased when these US strains were compared with other groups, such as European and Brazilian isolates [93]. Both aMPV-A and aMPV-B in the US show broader genetic connections and clear divergence when placed in a global context, highlighting how these viruses continue to evolve differently across regions worldwide.

### 4.2. Host Range and Susceptibility of aMPV

The common susceptible hosts for aMPV infection are turkeys and chickens, while birds of all age groups are vulnerable to infection with the aMPV. However, the disease is more severe in younger birds [4]. aMPV subtypes are adapted to cause infection in members of the order *Galliformes* (Table 3). Subtypes A, B, and C infect both turkeys and chickens, and subtype C of Eurasian lineage is well known to cause disease in ducks [24,26,32,36,74,94]. A recent study utilizing the aMPV-B/chicken/USA/SD-24/P6 demonstrated high pathogenicity in both chickens and turkeys with no age or host specificity [95]. This suggests the virulent nature of the virus, indicating the capability to adapt to multiple host ranges. aMPV-C was first isolated from pheasants in South Korea, where complete genome sequencing was also performed [39]. The existence of aMPV antibodies was detected in both reared and free-living pheasants in Italy, too, highlighting pheasants as a susceptible host [96]. Additionally, aMPV was identified in guinea fowl with respiratory signs, and the detected strain was classified as subtype B [97]. aMPV-C is known to cause disease in ducks, and its presence has been detected in Muscovy ducks in China and France [26,98], as well as in Sheldrake and Cherry Valley ducks in China [50,99]. aMPV-C was detected in wild birds (American crows, Cattle egrets, American coots, Canada geese and Rock pigeons) in Ohio, Arkansas, South Carolina and Georgia, highlighting the potential involvement of wild birds in introducing infection [69]. In addition, RT-PCR confirmation of aMPV RNA in several other wild species in the US, including Canada geese, blue-winged teal, sparrows, swallows, and starlings, further supports the contribution of wild birds in virus spread [100,101]. This implies the need for strict biosecurity control and identifies wild birds as the potential carriers of the novel US strain seen in 2023–2024. Moreover, aMPV subtypes A and B have also been reported in wild birds in Brazil, reinforcing that multiple subtypes can circulate across diverse wild avian populations [102,103].

**Figure 3:**
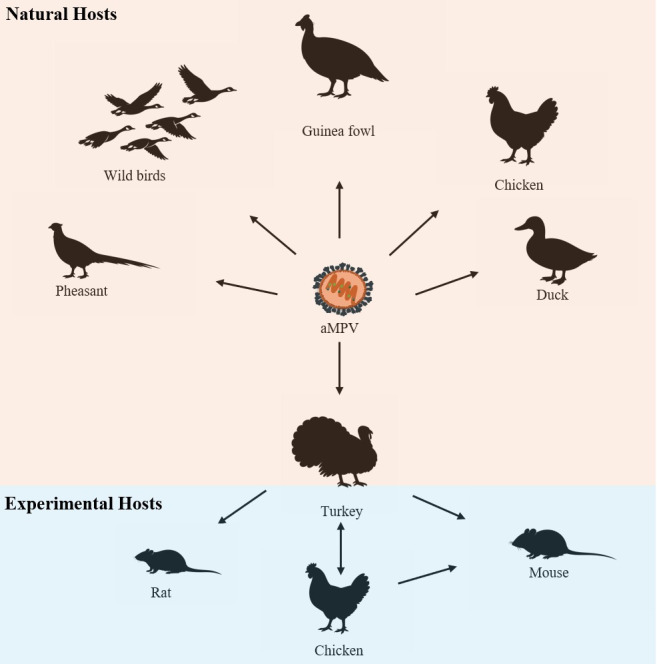
The host range for all aMPV subtypes (A, B, C and D). aMPV has been isolated from multiple species, including pheasant, wild birds, guinea fowl, chicken, and ducks. Experimental infection has demonstrated the ability of turkey-isolated aMPV to spill over in mice, rats, and bidirectionally transmit in chickens.

In an experimental study, it was demonstrated that aMPV subtypes A, B, C and D were all capable of infecting turkeys and chickens, with infection most severe in turkeys. And the duck origin aMPV-C strain was able to be isolated and induced seroconversion in both turkeys and chickens [94]. A novel subtype of aMPV was detected in gulls (American herring and Great black-backed), and these viruses appear to be intermediate between aMPV-C and the subgroup of other aMPV strains [104]. Similarly, another novel strain of aMPV was isolated from monk parakeets, and its sequence divergence from known strains also points to a new subgroup of aMPV [105]. Future studies aimed at understanding the pathogenic potential of these strains in domestic birds are essential for determining their impact on poultry production. A serosurvey in Zimbabwe detected aMPV antibodies in ostriches, demonstrating a 100% seroprevalence within the sampled population [106]. Experimental host range studies demonstrated that aMPV-C could be detected in mice for up to 14 days and in rats mainly at 4 days after inoculation [107]. Similarly, in an experimental study, an aMPV-C strain isolated from chickens in China was replication competent and persisted in the lungs of BALB/c mice for 21 days, showing lung lesions, fever, and upregulation of pulmonary inflammatory cytokines and chemokines. The aMPV-C isolate used in this study shared 78.5% similarity with hMPV, and although replication was observed in mice, no information is available regarding the common or potential receptors utilized by the virus [108]. These findings indicate that mice and rats may serve as potential carriers of aMPV and contribute to its transmission. aMPV has a broad and expanding host range, spanning domestic poultry, wild birds, and even non-avian species (Figure 3). This highlights the adaptable nature of aMPV and reinforces the importance of continued surveillance to prevent spillover events and protect poultry health.

## 5. Transmission Routes and Environmental Persistence

**Figure 4:**
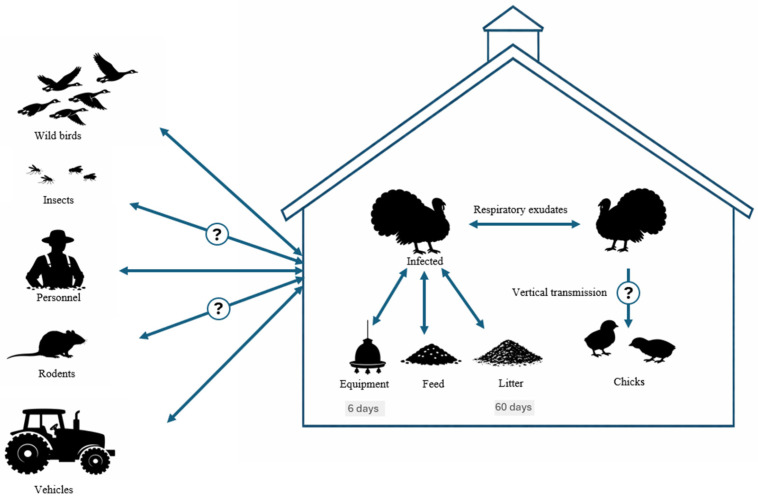
Transmission routes of aMPV. Survival studies on inanimate farm objects have shown that aMPV can remain viable on these materials for up to six days and can persist in litter for up to sixty days at −12 °C, highlighting the potential for indirect transmission via contaminated fomites [109,110]. The symbol “?” indicates that the transmission route has been suspected but has not been proven yet.

aMPV is primarily transmitted horizontally through infectious aerosols released by infected birds (Figure 4). Direct contact with respiratory secretions from infected birds through the oculonasal route represents the most common pathway of infection [4,18,19]. Studies utilizing subtype A and C have demonstrated direct contact transmission between birds; however, virus spread to birds housed in separate pens within the same room could not be established [18,19]. Even though the inoculated birds in these experiments became infected and transmitted the virus to birds in direct contact, birds in indirect contact did not develop disease, which highlights the difficulty of reproducing field-like transmission scenarios under laboratory conditions. Under experimental conditions, aMPV could be identified in the upper respiratory tract of birds for only a few days (6 or 7 days at most), indicating a short shedding period, yet infection still spreads rapidly within flocks and between farms under field conditions [14,18,19]. Aside from direct contact with infected respiratory secretions, other routes of infection remain unclear.

In addition to direct exposure to infected respiratory secretions, several indirect routes of infection, such as contaminated feed, water, litter, personnel, equipment, and vehicles, may also contribute to virus spread. Virus shed through respiratory secretions can contaminate these sources, allowing them to act as fomites for disease transmission [4,109]. Experimental studies investigating aMPV survivability in litter indicate that the virus can remain viable for up to 60 days under low temperature conditions [109]. Similarly, the survivability of aMPV on commonly encountered farm surfaces has been evaluated, and the virus was found to persist for up to six days [110]. Together, these findings highlight the potential for indirect transmission through contaminated materials and equipment.

Wild migratory birds also represent a major source of viral introduction and transmission. Several studies have reported the existence of aMPV in wild birds, highlighting their probable role in virus transmission [69,100,102]. Notably, aMPV RNA isolated from wild birds has been documented to share 90–95% nucleotide sequence identity with viruses from domestic turkeys [101]. Similarly, aMPV-C isolates recovered from wild birds have been demonstrated to cause disease in domestic turkeys [111]. Phylodynamic and phylogeographic analyses tracing the origins of the aMPV-A and aMPV-B outbreaks beginning in 2023 indicate introductions from Mexican and Eastern Asian strains respectively, further supporting the role of wild bird migration in viral spread [66]. The contribution of wild birds to the epidemiology and transmission of the disease in commercial turkey flocks is still not fully understood. Experimental studies have demonstrated the existence of the virus in the reproductive tract of layers, but there are still no findings consistent with vertical transmission to offspring [112,113]. aMPV-C isolated from turkeys and subsequently inoculated into ducks remained detectable in tissue samples for up to 21 days without causing any clinical signs, indicating that ducks can act as potential sub-clinical carriers capable of transmitting the virus [114]. The probable routes of introduction of aMPV-A and aMPV-B into US poultry remain poorly defined, and there is a need to expand investigations to include the potential roles of rodents and insects as mechanical vectors.

Generally, the virus is sensitive to external agents; however, lower temperature conditions favor the persistence of aMPV in the environment [115]. Experimental studies have demonstrated that aMPV is viable for more than 26 weeks at −70 °C and −20 °C, for less than 26 weeks at 4 °C, for less than 4 weeks at 20 °C, for 2 days at 37 °C, and for less than 6 h at 50 °C. aMPV was unaffected by variable pH for 1 h between pH 5 and 9, indicating that low pH environments could pose challenges for disposing of infected litter, with contaminated lakes potentially serving as sources of infection due to viral survival at lower pH levels. Common disinfectants such as quaternary ammonium compounds, ethanol, iodophors, phenolic derivatives, and sodium hypochlorite have been shown to reduce viral viability [115].

## 6. Pathogenesis and Immune Modulation

### 6.1. Clinical Presentation of aMPV Infection in Turkeys and Chickens

aMPV primarily targets the upper respiratory tract of susceptible birds following exposure to infected respiratory secretions, resulting in an acute respiratory infection. Clinical signs develop rapidly, with an incubation period ranging from 3 to 7 days, causing more severe infection in turkeys than in chickens, with the greatest severity observed in turkeys of 3 to 12 weeks of age [1,14,116]. The greater susceptibility of turkeys to the disease may be due to differences in viral replication rates and innate immune regulation between the two species [117]. The disease is referred to as turkey rhinotracheitis (TRT) in turkeys, and the symptoms include sneezing, coughing, tracheal crackles, nasal and ocular secretions, swollen infraorbital sinuses, and submandibular edema [1,4,116]. In chickens, in addition to lethargy, coughing, nasal mucus accumulation, and serous ocular exudates, swollen head syndrome (SHS), characterized by the edema of periorbital and infraorbital sinuses, has been associated with aMPV, which is often followed by cerebral disorientation, torticollis, and opisthotonos due to secondary *E. coli* infection [1,4,118]. In both turkeys and chickens, secondary infections caused by pathogens including *Ornithobacterium rhinotracheale*, *Bordetella* spp., *Pasteurella multocida*, *Staphylococcus* spp., *Mycoplasma gallisepticum*, Newcastle disease virus, infectious bronchitis virus (IBV) and infectious laryngotracheitis virus can exacerbate disease severity [4,16,17]. aMPV can also infect the reproductive tract (oviduct) of both laying hens and turkeys, resulting in poor shell quality, egg peritonitis, and reduced egg production, which in turn contributes to economic losses [112,113].

### 6.2. Pathogenesis and Tissue Tropism of aMPV

Following exposure to infected respiratory secretions, aMPV infects the ciliated epithelial cells of the upper respiratory tract, including the lining of cranial pneumatic bone air spaces and the nasolacrimal duct, causing ciliostasis and potentially resulting in total ciliary loss [119,120]. aMPV employs three proteins for cell entry and membrane fusion: G, SH and F protein. The G protein is accountable for virus binding to epithelial cells, whereas the SH protein contributes to cell-to-cell fusion. However, the F protein is capable of binding to cellular receptors and facilitating fusion of the viral and host cell membranes without requiring the involvement of the G or SH proteins, initiating virus infection [63,121,122]. For aMPV, the F protein exhibits dual functionality by mediating both receptor binding and membrane fusion, suggesting that fusion can occur independently of the attachment proteins [123,124]. The receptors for the aMPV F protein are not yet fully understood. Recent studies have indicated that integrins, a family of transmembrane cell adhesion molecules, can act as functional receptors for aMPV. In aMPV-C, integrin β1 has been identified as the receptor, whereas in aMPV-B, integrin αvβ1 has been shown to function as the receptor [121,124]. Viral replication occurs within the cytoplasm of the ciliated epithelium in both the respiratory and reproductive tracts [5,125].

**Figure 5:**
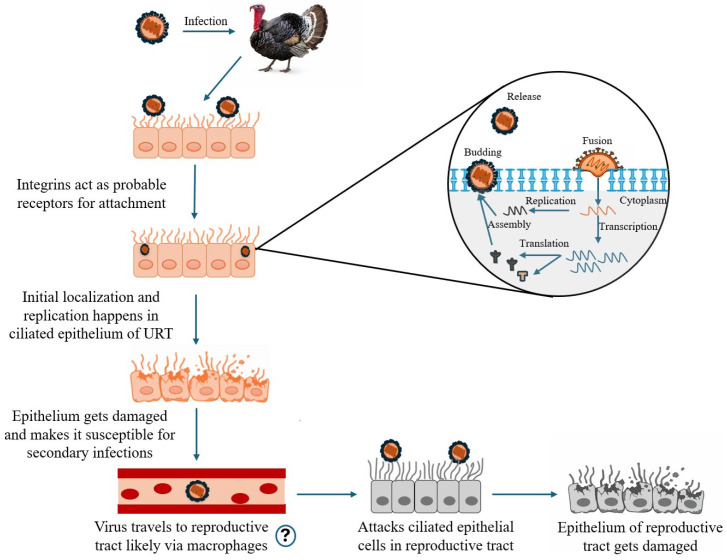
Schematic representation of the pathogenesis of aMPV. The symbol “?” indicates that this pathway has been suspected but has not been proven yet.

After infection, viral replication occurs within the ciliated epithelial cells of the upper respiratory tract. Experimental studies in turkey poults inoculated by aMPV-A through the ocular route have shown that high levels of virus can be recovered from the upper respiratory tract (nose and trachea) for up to 5 days post-infection, indicating an early viral response [19]. Histopathologic changes, including epithelial exfoliation, enhanced glandular secretion, epithelial deciliation, and mild infiltration by mononuclear cells, were observed in the nasal turbinates of both 3-week-old turkeys and chickens on days 1 and 2 post-infection with aMPV-B in an experimental study [120]. In another study using chicken and turkey tracheal organ cultures (TOCs), loss of ciliary activity was witnessed at 48 h post-infection (hpi) with aMPV-A in turkey TOCs and at 96 hpi in chicken TOCs [117]. Immunohistochemistry frequently reveals viral antigen along the apical membrane and ciliary border of infected epithelial cells, highlighting aMPV’s strong affinity for ciliated epithelium [117,125]. These findings highlight that aMPV rapidly damages the ciliated epithelium of the upper respiratory tract and compromises mucociliary clearance, facilitating further complications by secondary infections (Figure 5).

After a respiratory infection, the virus can disseminate systemically to the reproductive tract, leading to infection of those tissues. In a study on turkey poults with aMPV-C, viral antigen was identified in a small number of macrophages within the nasal turbinates and sinuses. This finding suggests that macrophage-mediated leukocyte trafficking could facilitate the virus’s movement to the reproductive tract [119,126]. The role of macrophages in aMPV infection needs to be studied in detail, as studies of other avian viruses, such as IBV, which targets avian macrophages, suggest that macrophage involvement may facilitate viral dissemination beyond the respiratory tract [127,128]. aMPV infection damages the ciliated epithelium of the reproductive tract in laying hens and turkeys, contributing to decreased egg production [112,113,119]. In an experimental study utilizing oviduct organ cultures (OOCs) infected with aMPV-A and aMPV-B, ciliary loss was observed beginning at 48 hpi, along with epithelial cell flattening and submucosal edema. These lesions highlight that the decline in egg output and formation of thin-shelled eggs might be attributed to aMPV infection instead of secondary infections [125].

### 6.3. Immune Responses to aMPV Infection

aMPV infection elicits strong innate and adaptive immune responses in susceptible hosts, involving both humoral and cell-mediated responses [129]. Turkey poults inoculated via the oculonasal route at 4 weeks of age developed a robust humoral response, with aMPV-C specific IgG antibodies first detected in serum samples at 7 days post-infection and persisting for up to 14 weeks [126]. Infiltration of IgA^+^ B cells into the upper respiratory tract after challenge with aMPV-A and aMPV-C has been confirmed, and similar infiltration by IgM^+^ and IgG^+^ B cells has also been documented [14,130,131]. However, studies have shown that humoral immunity alone does not provide effective shielding against aMPV infection and does not reliably reflect actual protective immunity against aMPV. Infection of turkey poults with aMPV-A after chemical treatment with cyclophosphamide, a B-cell suppressor, resulted in a lack of clinical signs, indicating the implication of cell-mediated immunity [132]. Similarly, reduction in T-lymphocytes using cyclosporin A, followed by infection with aMPV-A in turkeys, resulted in prolonged resolution of clinical signs and lesions, as well as positive choanal swabs, demonstrating the importance of cell-mediated immunity [133].

Experiments in turkeys have demonstrated an increase in CD4^+^ T cells induced by both virulent and vaccine strains of aMPV-A and aMPV-B at 7 and 14 days post-infection in the Harderian gland and spleen. In chickens, CD4^+^ T cells increased fourfold at 6 days post-inoculation (dpi) and a concurrent rise in CD8α^+^ cells was observed in the Harderian gland at 6 dpi [129,134]. CD8^+^ T cells are known to contribute to viral clearance in respiratory infections through contact-dependent effector mechanisms (perforin and CD95L) as well as the production of IFN-γ and TNF-α. Therefore, the stronger CD8α^+^ (CD8^+^ T-cell and natural killer cell) response in the Harderian gland of broilers likely contributes to their faster viral clearance and recovery compared with turkeys [134,135]. In these same studies, IFN-γ and IL-6 levels were upregulated in turkeys in the spleen cells and Harderian gland following infection with aMPV-A and aMPV-B, beginning at 3 and 5 days post-infection, respectively. In chickens, IFN-γ upregulation was observed in the Harderian gland after aMPV-A infection, with increased expression also detected in the nasal turbinates at 3 dpi. Even though IFN-γ levels were upregulated in the nasal turbinates of chickens infected with aMPV-B, the levels were downregulated in the Harderian gland at 3 dpi [129,134]. In studies utilizing turkey and chicken TOCs, induction of apoptosis was evaluated following aMPV infection. Apoptotic cells were not discovered in turkey TOCs, whereas modest counts were observed in chicken TOCs at 96 and 168 hpi, correlating with the onset of epithelial cell loss. Histological examination of turkey TOCs revealed necrosis and degeneration of epithelial cells, suggesting that epithelial cell loss in turkey TOCs may be attributable to necrosis [117]. IFN-α mRNA expression was downregulated in both TOCs, with a non-significant upregulation observed only in chicken TOCs at 192 hpi. This may explain why aMPV-infected turkey TOCs did not exhibit increased apoptosis, despite IFN-α being a known inducer of apoptotic pathways [117]. There was also an upregulation of iNOS in both TOCs, which is known to be expressed in macrophages and epithelial cells. The sustained upregulation up to 120 hpi in chicken TOCs may also indicate a correlation with the apoptosis observed in chicken TOCs [117]. These findings help describe the greater vulnerability and more serious clinical signs observed in turkeys, both in TOC studies and under experimental trials and field observations. In a study on aMPV-C, the P protein was shown to reduce IFN-β production and the interferon stimulated genes (ISGs) expressions. Interferon regulatory factor 3 (IRF3) was identified as the target of the P protein, which inhibits IRF3 translocation from the cytoplasm to the nucleus, thereby suppressing IFN-β induction [136]. Similarly, aMPV-C was shown to induce reduction in mitochondrial antiviral signaling (MAVS) protein in Vero cells, which is crucial for linking upstream viral RNA recognition to downstream signaling pathways [137]. These studies highlight immune evasion mechanism employed by aMPV, underscoring its ability to modulate host antiviral signaling.

## 7. Diagnostics and Surveillance

Various diagnostic tools have been established over time to enable the reliable detection of aMPV. Diagnosis centered solely on clinical signs and gross lesions is challenging, as many respiratory diseases present similar clinical observations. Thus, differential diagnosis is needed as paramyxoviruses such as Newcastle disease virus and avian paramyxovirus-3, as well as IBV, influenza viruses, and multiple bacterial and *Mycoplasma* species, can produce similar clinical signs [4,119]. Therefore, confirmatory laboratory testing is required to establish a definitive diagnosis of aMPV. RT-PCR serves as the gold standard for aMPV diagnosis because it enables rapid and sensitive molecular detection, whereas virus isolation also serves as a gold standard by providing confirmation through recovery of live, infectious virus. However, due to the difficulty and low success rate of isolating aMPV, RT-PCR is the method most commonly used in routine diagnostic practice [47,138,139]. aMPV is shed for only a short period, with the highest likelihood of virus isolation occurring around 5–7 days post-infection, coinciding with the appearance of clinical signs. Beyond this point, isolation and detection become difficult, underscoring the critical importance of sampling time [18,19,113]. The most suitable samples for aMPV detection are obtained from ocular or nasal secretions, or from turbinate, sinus and tracheal tissues [4,138,140]. The commonly used methods for the definitive diagnosis of aMPV include:

### 7.1. Virus Isolation

Virus isolation can be performed using embryonated eggs, TOCs and cell culture systems. The initial successful isolation of aMPV in both South Africa and the United States was carried out in embryonated eggs using the yolk sac inoculation route [6,138]. Six to eight-day-old embryonated turkey and chicken eggs are typically used for inoculation via the yolk sac route. Hemorrhages and some embryo mortality may be observed as lesions. Serial passaging is needed to induce embryo death, making the procedure longer; however, highly reliable [6,138].

TOCs is the commonly performed system in today’s date for aMPV isolation. Early studies in South Africa achieved isolation using tracheal ring organ cultures from 27-day-old turkey embryos, subjected to sinus exudate collected from turkeys exhibiting rhinitis and sinusitis [6]. TOCs can be maintained for several weeks, and ciliostasis (cessation of ciliary beating) is the primary characteristic typically appearing around 5–7 days post-inoculation [6,32,138]. Cell cultures are used for aMPV multiplication, although they are often unreliable for primary isolation. Following initial isolation in embryonated eggs or TOCs, the virus can adapt to replicate in several cell culture systems, involving chicken embryo fibroblasts (CEF), chicken embryo liver cells (CEL) and Vero cells (Table 4) [141,142]. Vero cells (derived from African green monkey kidney epithelial cells) lack type I interferons [143]. A characteristic cytopathic effect, typically featuring syncytial formation, becomes evident within seven days after the virus adapts to cell culture [4,116,138]. In addition, an experimental study demonstrated that chicken embryo related (CER) cells can support the primary isolation of aMPV subtypes A and B, and that CER, Vero and BHK-21(baby hamster kidney) cells are capable of propagating these subtypes to high viral titers [144]. Compared with these cell lines, Vero cells exhibit more pronounced syncytia formation, likely due to the absence of type I interferon response [54,116].

### 7.2. Molecular Detection

Molecular identification methods, particularly RT-PCR and RT-qPCR, are the most widely used for aMPV detection because of their speed, high sensitivity, and specificity. Turbinates, choanal, and tracheal swabs are appropriate sample types for RT-PCR-based detection of the viral genome [14]. Due to the presence of distinct aMPV subtypes, most RT-PCR assays are designed to be subgroup-specific and therefore do not detect all subtypes in a single reaction. RT-PCR was first utilized to detect the F protein gene in six-week-old turkey poults inoculated with the virulent UK/3B/85 strain [63,151]. In a subsequent study comparing F-, G-, and N-based RT-PCR protocols, the results demonstrated that RT-PCR using N-gene primers exhibited broader specificity than the F- or G-based assays for diagnostic purposes, and the G-gene-specific RT-PCR primers enabled subtype identification. These findings highlight that N- and G-based RT-PCR assays are useful for rapid detection and early subtyping of aMPV in field samples, respectively [12]. The use of subtype-specific RT-PCR assays at the outset can result in other circulating subtypes being overlooked. Therefore, using an N-gene-based RT-PCR first for broad detection of aMPV, followed by subtype-specific PCRs or sequencing for accurate typing would be more appropriate [4,12,138]. In a study conducted in China, a quadruplex real-time RT-PCR assay was developed using four pairs of specific primers and four corresponding probes targeting the G or M genes of all aMPV subtypes, providing highly specific and highly sensitive detection of all aMPV subgroups [152]. Similarly, a five-plex digital droplet RT-PCR assay that targets the drift-resistant viral polymerase (a conserved gene of aMPV) was developed and successfully identified all four aMPV subgroups, whose principle relies on changing probe concentrations [153]. Next-generation sequencing of samples that tested positive by RT-qPCR was used to differentiate aMPV strains during the recent aMPV subtype A and B outbreak in the US [8,119]. The absence of a universal and extensively validated primer set targeting a truly conserved region of the aMPV genome across different molecular platforms represents a significant gap in current diagnostic capabilities. Developing such primers would be essential for reliable molecular detection and for accurately assessing viral distribution across regions.

### 7.3. Serological Tests

Serological methods are widely used to verify aMPV infection in commercial poultry and additional avian species because virus isolation and direct detection are often difficult. Serological techniques such as ELISA, virus neutralization tests, and indirect immunofluorescence are utilized, among which ELISA is the most widely applied. These assays are better suited for flock-level screening and surveillance than for definitive diagnosis [4,14].

Various commercial ELISA kits are available for the serological detection of aMPV. Most ELISA assays use the indirect format, while some employ a blocking (competitive) format. In the indirect ELISA format, microtiter plates coated with aMPV antigen are incubated with test serum and subsequently with an enzyme-labeled anti-turkey or anti-chicken conjugate. The measured enzyme activity correlates with the concentration of specific antibodies in the serum, and aMPV-specific antibodies are indicated by the development of color. Conversely, the blocking (competitive) ELISA functions by allowing antibodies in the test serum to compete with a labeled antibody for available antigen-binding sites [138]. In one study, a competitive ELISA based on the ability of serum antibodies to compete with a monoclonal antibody for binding to recombinant aMPV N protein, which was used as antigen, was developed. This assay demonstrated a specificity of 100% and a sensitivity of 98% when compared with the virus neutralization test. Notably, while the commercial ELISA kit detected aMPV antibodies only after 10 days post-infection (PI), the competitive ELISA was able to sense antibodies as early as 5 days PI [154]. For early diagnosis of aMPV infection to be successful, the choice of ELISA antigen is critical, as the sensitivity of the assay depends heavily on the antigen used [155]. In one study, ELISAs that used a subgroup A or B as antigen were able to detect antibodies to aMPV subtypes A and B but failed to detect antibodies against aMPV-C, demonstrating that antigen choice can significantly influence assay performance [7,138].

Virus neutralization tests are used less frequently than ELISA for diagnosing aMPV infections. They can be performed in several systems, including TOCs, tissue culture systems such as Vero cell monolayers, and CEFs [138]. Although virus neutralization is costly and not easily scalable for surveillance of large flock populations, it can be employed to corroborate and validate ELISA findings [119]. Indirect immunofluorescence and immunodiffusion techniques may likewise be performed on tissue sections to identify antibodies against aMPV [156].

## 8. Vaccines

aMPV infection cannot be controlled with medication; therefore, disease prevention relies primarily on strict biosecurity measures and vaccination. Inactivated and live attenuated vaccines are present for both turkeys and chickens. In earlier periods, the development of suitable vaccines was hindered by the lack of consistent challenge models needed to conduct vaccine-efficacy studies. However, recent studies have demonstrated effective attenuation of aMPV strains in various cell culture systems and their beneficial use as vaccine candidates [4,157,158]. Effective aMPV vaccines must induce robust cell-mediated and mucosal immunity, since humoral responses by themselves do not provide full protection and are poor predictors of vaccine performance [159].

### 8.1. Live Attenuated Vaccines

Live attenuated vaccines constituted the main approach for outbreak control in the early years of aMPV management in Europe. These attenuated vaccines used in Europe were developed from European aMPV strains that had been attenuated through serial passage in embryonated eggs, Vero cells, or TOCs [160,161]. In Europe, subtypes A and B were used to produce live attenuated vaccines, whereas in the United States, subtype C was used. Live attenuated vaccines may be delivered via coarse spray, aerosolization, drinking water, or oculonasal application, all of which aim to stimulate strong mucosal and cell-mediated immune responses in the respiratory mucosa. Protection against aMPV clinical signs and lesions equivalent to that induced by the oculonasal route was achieved when the vaccine was delivered by spray or through drinking water, methods that are more practical for large-scale field use [5,156,162].

In turkeys, vaccination with either subtype A or subtype B strains provides substantial cross-protection against both subtypes. Similarly, studies in day-old broiler chickens have demonstrated that vaccination with aMPV subtype B can induce protection not only against subtype B but also against subtype A [155,163,164]. A study reported that subtype A and B vaccines conferred protection in turkeys challenged with aMPV-C, while an aMPV-C-derived vaccine conferred protection in turkeys and somewhat worked in chickens against aMPV-A but failed to protect against aMPV-B [7]. These results indicate that although cross-protection can occur among certain aMPV subtypes, its inconsistency reinforces the need for subtype-specific vaccines to ensure dependable protection in the field.

Even with effective live attenuated vaccines in place, disease outbreaks persist in vaccinated flocks. This can result from reversion to virulence, whereby the vaccine virus acquires mutations during replication in birds and regains pathogenic characteristics. A live attenuated subtype A vaccine was shown to revert to virulence after only two coding mutations and was capable of infecting day-old turkey poults [165]. Farm-level spray administration can lead to uneven vaccine delivery, leaving some birds with suboptimal doses and others completely unvaccinated. Because vaccinated birds can shed aMPV, these unvaccinated flock mates may become infected by the shed vaccine virus, creating conditions that increase the likelihood of reversion to virulence [165]. Similar findings were reported for a subtype B live attenuated vaccine, which also reverted to virulence and caused disease in day-old poults comparable to that produced by field strains [166]. A turkey farm in Italy that had been vaccinated against aMPV-B became infected with aMPV-A, and sequencing showed that the virus originated from a vaccine strain that had not been used on the farm for at least six months, nor in any farms within a 5 km radius [167]. These outcomes indicate that reversion to virulence of live vaccines is a major concern and demonstrate how vaccine-derived virus can even introduce disease into farms where the pathogen was previously not present. Another major factor influencing vaccine efficacy is the presence of maternally derived antibodies (MDA). The higher levels of MDA can result in a lower protective effect from the live attenuated vaccine. In an experimental study in turkeys, vaccination with an attenuated aMPV-A strain induced increased CD8^+^ T-lymphocyte responses in the Harderian gland and tracheal mucosa of MDA-negative birds, whereas in MDA-positive birds the response was dominated by CD4^+^ T cells in these tissues. Similarly, vaccination elicited increased anti-aMPV IgY antibodies in MDA-negative birds, while in MDA-positive birds, the decline in serum MDA antibody levels occurred more rapidly, likely due to immunophagocytosis [168]. Together, these observations underscore the need to consider maternal antibody status when designing effective vaccination programs in young birds.

### 8.2. Inactivated Vaccines

Inactivated oil emulsion or water-based adjuvanted vaccines incorporating subtypes A and B are commonly employed as part of infection control strategies. In Europe, a commercially available inactivated oil-emulsion vaccine is widely used in both turkeys and chickens. Inactivated vaccines are generally administered as booster doses following live attenuated vaccines [14,156]. It was shown that administering an inactivated subtype A aMPV vaccine at 30 weeks of age, after priming with a live attenuated subtype A vaccine at 1 week of age, provided strong protection of laying performance when birds were challenged at 38 weeks of age [169]. Two-week-old turkeys inoculated oculonasally with an inactivated aMPV-C vaccine adjuvanted with the synthetic double-stranded RNA polyriboinosinic polyribocytidylic acid showed elevated mucosal IgA^+^ cells in the upper respiratory tract, along with enhanced virus-specific IgG and IgA in the lachrymal fluid and the serum IgG. Vaccinated birds were protected against challenge and exhibited reduced respiratory lesions when challenged at either 7 or 21 days post-vaccination [170]. In Egypt, intramuscular administration of an inactivated aMPV vaccine prepared from the GIZA TRT-4 strain (subtype B) and adjuvanted with *Nigella sativa* oil in 21-day-old turkey poults elicited superior humoral and cell-mediated immunity relative to commercially available inactivated or live vaccines [171]. Similarly, in China, an inactivated aMPV-B vaccine combined with a new adjuvant comprising immune-stimulating complexes (ISCOMs) administered intramuscularly to 21-day-old SPF chickens induced high levels of virus-specific and virus-neutralizing antibodies. It also stimulated B- and T-lymphocyte responses and upregulated the IL-4 and IFN-γ levels [172]. Together, these results highlight the importance of inactivated vaccines, as it reduces the chances of shedding the live virus into the environment, and also decrease the likelihood of reversion to virulence pathogen.

### 8.3. Other Types of Vaccines

The fusion protein of aMPV-A was utilized to develop the recombinant fowl pox virus vaccine when administered to one-week-old turkey poults intramuscularly and by wing-web at a two-week interval, resulting in milder clinical signs and a 1000-fold reduction in challenge virus recovered from the nose and trachea, demonstrating that the F protein plays a major role in protection against aMPV [173]. Similarly, DNA vaccination using plasmids encoding the F gene (pCMV-F) from aMPV-C produced comparable results, as intramuscular administration to one-week-old turkey poults induced significant protection [174]. Additionally, a DNA vaccine using the plasmid pGEM-T-Easy vector to express the F gene from aMPV-B, administered intramuscularly to 21-day-old turkey poults, provided 100% protection in the vaccinated birds [175].

Through a reverse-genetics approach, a bivalent recombinant vaccine was formulated using the glycoprotein of aMPV-C in a LaSota strain as a backbone. SPF turkey poults (sixty-one-week-old), inoculated intranasally and intraocularly, developed incomplete protection against pathogenic aMPV-C and complete protection against velogenic NDV, underscoring that a single aMPV-C G protein is inadequate for comprehensive protection against aMPV-C [176]. Similarly, NDV-derived recombinant vector vaccine expressing the F and G genes of aMPV-C worked substantially better, providing protection against pathogenic aMPV-C challenge, while complete protection was reported against velogenic NDV [177].

As of April 2026, the US poultry industry is actively using emergency import licenses and experimental autogenous vaccines to combat aMPV subtypes A and B. While no fully licensed, domestically manufactured vaccines were traditionally available for the recent outbreaks, the USDA now permits the import and use of specific products like RESPIVAC^®^ aMPV (modified live vaccine, aMPV-B), HIPRAVIAR^®^ TRT (inactivated vaccine, aMPV-B), Vaxxon^®^ SHS (live vaccine, aMPV-B), Poulvac^®^ TRT (modified live vaccine, aMPV-A) and Boehringer-Ingelheim products: NEMOVAC^®^ (modified live vaccine, aMPV-B), AVIFFA RTI (modified live vaccine, aMPV-B), TUR-3 (inactivated vaccine, aMPV-B) to mitigate severe economic losses (Table 5) [47,71,178]. To support outbreak control within the US, Merck Animal Health, Cambridge Technologies and Ceva have begun producing experimental autogenous vaccines using subtype B isolates sourced from domestic poultry flocks. In addition, Vaxxinova USA is producing an experimental autogenous vaccine based on a US-origin subtype A isolate [47,71].

The USDA issued the first-ever permit for the importation of a modified live vaccine produced outside the US on 20 December 2024, and the vaccine approved under this authorization was Vaxxon^®^ SHS [71,179]. The importation permit of this live vaccine into the US for the first time highlights the critical importance of controlling aMPV in the country, given that US regulatory requirements for vaccine importation are typically stringent and only done when no other options are available. Despite the permit for vaccine importation in 2024, aMPV cases continued to rise, with 13.3 million cases reported in 2025. This may indicate setbacks in the implementation of these vaccines across the US, or it may reflect limitations in the effectiveness of the imported vaccine under field conditions. Reversion to virulence is a risk associated with the use of live aMPV vaccines and has been reported in other countries [165,166]. Relying on live vaccines derived from European strains may also increase the risk of introducing vaccine-derived viruses into the US through reversion to virulence, and these vaccines could also be less effective against US-origin strains [167]. Therefore, the use of killed or inactivated vaccines may be more advantageous, and evaluation of vaccine performance is critical to prevent the circulation of potential vaccine-derived strains.

## 9. Industry Relevance

In the US poultry industry, aMPV has recently emerged as an economically important priority pathogen, causing major losses in turkeys, broilers, and commercial operations. aMPV infections result in substantial economic losses, reduced production efficiency, and broader market-level disruptions. Turkey breeders experience egg production losses ranging from 20% to 80%, lasting 2 to 4 weeks, resulting in a shortage of poults. Similarly, broiler breeders exhibit a moderate decline in egg production, typically ranging from 5% to 10%. Secondary infections with pathogens such as *E. coli*, *Ornithobacterium rhinotracheale*, and *Mycoplasma gallisepticum* further complicate the clinical disease, increasing mortality and adding to overall economic losses [17,71,119]. Together, these losses highlight the growing economic threat posed by aMPV and its significance as a priority pathogen for the US poultry sector.

In the recent US Animal Health Association (USAHA) annual turkey health survey, aMPV rose dramatically from its previous position at #38 in 2023 to the #1 reported issue in 2024 with 2355 reported cases [71]. This rapid change in ranking underscores the high transmissibility of aMPV, its capacity to cause substantial production losses across multiple poultry species, and the current absence of effective therapeutic options.

Historically, even in areas where aMPV vaccination is regularly practiced, the virus has been considered the most important respiratory pathogen of turkeys aside from avian influenza. Even during the initial aMPV-C outbreak in the US, the Minnesota turkey industry faced serious economic losses, estimated at approximately $15 million per year from 1997 to 2002 [4,180]. The 2024 report by the Minnesota Turkey Growers Association reported that the Minnesota turkey industry experienced $112 million in lost sales, a $17 million reduction in labor income, $31 million in lost value added to the state’s economy and nearly $8 million in reduced tax revenue. An estimated 2,201,903 turkeys were lost to aMPV in 2024, representing approximately 6.57% of Minnesota’s annual production [15]. These early 2024 outbreaks have led to increased diagnostic testing, biosecurity changes, production delays and heightened supply chain demands, further contributing to the economic burden.

The economic relevance of aMPV goes beyond farms into animal health and vaccines. The global aMPV treatment market, composed primarily of vaccines, is valued at $311.6 million in 2025 and is projected to reach $579.5 million by 2035 [181]. Within this market, the vaccine segment dominates with a 68.4% share, reflecting the industry’s strong reliance on immunization for disease control. Overall, aMPV accounts for approximately 3–5% of the global poultry healthcare market [181]. These patterns show that aMPV is not only a production-level threat but also a major contributor to shaping the global poultry health and vaccine markets.

### Production, Economic Value and aMPV Disease Patterns

**Figure 6:**
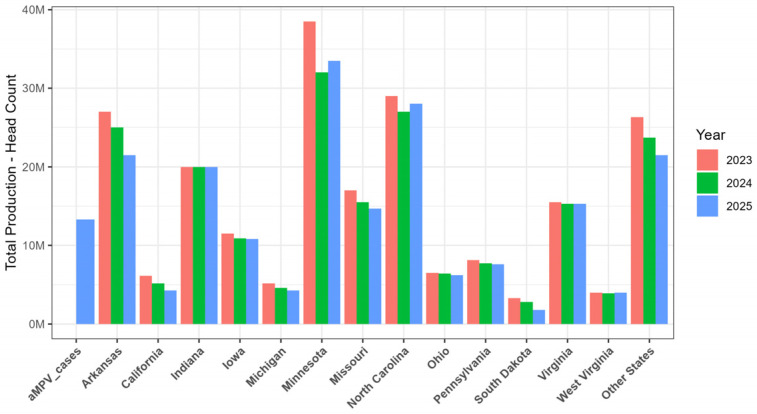
Turkey production across states from 2023 to 2025 based on head count. aMPV cases per head count in production turkeys are reported with 2355 cases in 2024 and 13.3 million cases in 2025. Raw data were collected from the US Department of Agriculture, National Agriculture Statistics Service [182].

The decline in turkey production between 2023 and 2025 is majorly associated with the aMPV outbreaks in major turkey-producing states. The escalation of turkey losses during this period coincides with the first US detections of aMPV-A and aMPV-B. US states such as Arkansas, California, Minnesota, Missouri and North Carolina clearly demonstrate a decrease in turkey production from 2023 to 2025. Importantly, the largest turkey-producing state in the US, Minnesota, declined from approximately 38.5 million birds in 2023 to about 32 million birds in 2024, followed by 33.5 million birds in 2025 (Figure 6). There were approximately 13 million birds impacted by aMPV in 2025, an increase from 2355 reported cases in 2024, which is interestingly higher than the total production in head count for states such as California, Iowa, Michigan, Ohio, Pennsylvania, South Dakota, and West Virginia. This refers to the potential capability of aMPV to eradicate the turkey production, emphasizing the need to invest in understanding aMPV transmission, pathogenesis and prevention. The slight recovery documented in the year 2025 may be attributed to the first import of the live aMPV vaccines for emergency use in turkeys and the enhanced biosecurity measures, which would have reduced the viral pressure. In the poultry industry, viral pressure is directly proportional to vaccination, biosecurity, movement control, flock immunity and reduction in the viral shedding from the infected birds. The reduction in the quantity of infectious virus present on farm surfaces, equipment, litter and other fomites, which can serve as sources of aMPV infection, helps lower the overall viral pressure in the environment. As the environmental viral load decreases, the likelihood of susceptible birds becoming infected also diminishes, thereby interrupting transmission within and between poultry flocks.

**Figure 7:**
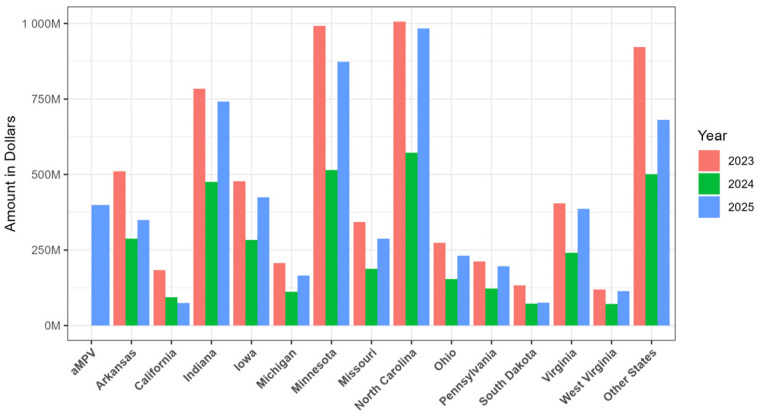
Turkey production across states from 2023 to 2025, based on the amount in dollars. aMPV led to approximately $44,000 loss in 2024 and $398 million loss in 2025. Raw data were collected from the US Department of Agriculture, National Agriculture Statistics Service [182].

The aMPV disease impact was devastating for the turkey industry, leading to a substantial decrease in the value of production in several states, including Arkansas, California, Indiana, Iowa, Michigan, Minnesota, Missouri, North Carolina, Ohio, Pennsylvania, South Dakota, Virginia, West Virginia and several other states (Figure 7). For instance, Minnesota’s production value in 2023 was nearly $1 billion, which dropped to $0.88 billion in 2025, suggesting a drop of 0.12 billion. An overall estimated economic loss of approximately $398 million is speculated for the year 2025 in the US, with $0.898 per pound for turkey meat and considering the average weight of live turkeys processed under federal inspection to be 33.33 pounds. Surprisingly, the overall aMPV-related loss is higher than the value of production in Arkansas, California, Michigan, Missouri, Ohio, Pennsylvania, South Dakota and West Virginia. This emphasizes the significant economic impact aMPV can have on the overall agricultural production system in the US.

There were a few other reported cases in turkeys during 2025, including Histomoniasis, *Mycoplasma synoviae*, Turkey coronavirus, Turkey reovirus, and *Mycoplasma gallisepticum*, all of which were documented only in the low hundreds [71].

## 10. Knowledge Gaps and Future Directions

Despite being recognized since 1978, a lack of study regarding aMPV limits our ability to fully understand and control the virus. The transmission routes of aMPV remain unclear, as direct contact is the only fully established mode of transmission. However, additional transmission routes appear to exist, but these remain unidentified and poorly characterized. Under experimental conditions, aMPV has been shown to be shed through respiratory secretions for only very short periods by infected birds, and as an enveloped virus, it is not highly resistant outside the host. Given these characteristics, the rapid spread of aMPV over considerable distances remains difficult to explain [183].

Another significant knowledge gap concerns the mechanisms underlying host–virus interactions. Although aMPV and hMPV share conserved polymerase and nucleocapsid functions, the host-specific adaptations that shape pathogenicity and immune evasion strategies are less understood for aMPV than for hMPV [5]. Despite clear evidence that aMPV can infect the reproductive tract, the mechanisms behind its dissemination from the respiratory mucosa to the reproductive tissues have not been characterized, representing a significant gap in our understanding of aMPV pathogenesis. Addressing these gaps will be essential for expanding ground-level surveillance systems, refining intervention approaches and ultimately reducing the global impact of aMPV.

Lack of specific therapeutics and antivirals against aMPV signifies the disease management as an strategical approach via enhanced biosecurity, supportive care and control of secondary bacterial infections using broad-spectrum antibiotics like enrofloxacin [184]. In an experimental study, subcutaneous administration of gamithromycin to turkeys was shown to exert beneficial effects against secondary *Ornithobacterium rhinotracheale* infection in turkeys previously infected with aMPV-A [185]. Currently, there is no specific antiviral drug that has been approved to be used against aMPV infection. In vitro studies utilizing plant-derived compounds have shown promising antiviral activity against aMPV. Extracts obtained from four plant species, *Gaylussacia brasiliensis*, *Arrabidaea chica*, *Virola sebifera* and *Aspidosperma tomentosum* were able to inhibit approximately 99% of aMPV-A in vitro [186]. Although further studies are needed to identify the specific active compounds responsible for the observed antiviral effects, such findings provide promising evidence for plant-derived interventions. RNA interference using short interfering RNA (siRNA) targeting the nucleoprotein (N) mRNA has been demonstrated to reduce viral titers in vitro, indicating a unique approach that could be further explored as a potential antiviral therapy [187]. Overall, the limited availability of antivirals against aMPV highlights a significant knowledge gap, making vaccination and strict biosecurity the only control measures that are practically applicable in the poultry industry.

Future directions in aMPV research should emphasize epidemiology, pathogenesis, host–virus interactions and control strategies. Routine surveillance to track viral evolution and transmission networks is crucial for advancing our understanding of aMPV epidemiology. Studies on viral entry, replication, immune evasion, receptor usage and host-specific adaptations are all critical for increasing our understanding of aMPV pathogenesis and for improving strategies for prevention and control. Diagnostic screening should be expanded to include all aMPV subtypes (A, B, C and D), particularly given evidence that subtype D is well adapted to infect both turkeys and chickens, making it surprising that only a single case of aMPV-D has been reported since the 1980s [94]. Understanding of the virulence genes of subtypes A, B, and C via comparison with D would allow us to formulate well-targeted inhibitory antivirals against the aMPV. The re-emergence and rapid detection of aMPV-A and aMPV-B in the US after a decade of absence underscores significant weaknesses in existing biosecurity measures, highlighting the need for strengthened and more consistently implemented biosecurity practices.

## 11. Conclusions

aMPV is a highly contagious, devastating and economically important pathogen of turkeys. The introduction of aMPV subtypes A and B in the US resulted in significant economic loss in the turkey and overall poultry industry, leading to the first-ever import and utilization of the live attenuated vaccine in the US agriculture. Research encompassing the host range, transmission routes, pathogenesis, vaccine and effective control measures is urgently needed to combat the aMPV disease in the poultry system. In addition, the impact of field strains in the turkey production systems still needs to be investigated for the formulation of broad treatment and prevention strategies against aMPV. Summarizing the aMPV research gives us a somewhat clear picture of the knowledge gaps in the aMPV literature. Addressing these gaps while emphasizing diagnostic surveillance and biosecurity needs will be crucial for mitigating future outbreaks and lowering the overall impact of aMPV on poultry health and production.

## Figures and Tables

**Table 1:** Countries with the presence of aMPV (“-” indicates unavailable data).

Country	First Detection (Year)	Domestic Host Species	Subtypes Detected	Sample Types	Detection Methods	References
South Africa	1978	Turkeys	A	Sinus exudate	Embryonated SPF eggs, tracheal-ring organ culture, electron microscopy	[6,22]
France	1981	Turkeys, chickens and ducks	B, C and D	Trachea, rhino-pharyngeal swabs	Embryonated SPF eggs, RT-PCR	[23,24,25,26]
Italy	1984	Turkeys, chickens and ducks	A, B and C	Blood samples, choanal cleft swab	Indirect ELISA, RT-nested PCR	[27,28,29]
United Kingdom	1985	Turkeys and chickens	A and B	Swabs from the esophagus, trachea, nasal turbinate, pharynx	Virus neutralization tests with mouse monoclonal antibodies and polyclonal antisera, RT-PCR	[30,31,32,33]
Germany	1987	Turkeys and chickens	A and B	Nasal exudate, trachea	Nested RT-PCR	[34]
Hungary	1989	Turkeys	B	-	Virus neutralization tests with mouse monoclonal antibodies and polyclonal antisera, RT-PCR	[31,35]
Japan	1989	Chickens	A and B	Trachea	RT-PCR	[36]
South Korea	1992	Chickens and pheasants	A, B and C	Oropharyngeal and tracheal swab, nasal turbinate	Multiplex RT-PCR, RT-PCR	[37,38,39]
Malayasia	1994	Chickens and quails	A and B	Oropharyngeal, cloacal and tracheal swab, turbinate and lung tissues	RT-PCR, ELISA	[40,41,42]
Brazil	1995	Turkeys and chickens	A and B	Nasal turbinate, sinus, lung and tracheal tissues, oviduct and testis tissues (in cases with reproductive issues)	RT-PCR	[43,44]
United States	1996	Turkeys and chickens	A, B and C	Nasal turbinate and tracheal tissues, tracheal swabs, serum samples	Virus isolation in Vero cells, chicken embryo fibroblast cells, immunofluorescence staining, RT-PCR, ELISA	[7,45,46,47]
Chile	1997	Turkeys and chickens	B	Serum samples	ELISA	[48]
Belgium	1998	Turkeys	A and B	Serum samples, tracheal swabs	Direct immunofluorescence technique, serum neutralization test	[49]
China	1999	Turkeys, chickens and ducks	A, B and C	Nasal turbinate, oviducts, follicles and air sacs, tracheal swabs	Electron microscopy, RT-PCR, and indirect immunofluorescent antibody (IFA) analysis	[50,51,52]
Jordan	2004	Chickens	B	Nasal turbinate	Competitive ELISA, RT-PCR	[53]
Mexico	2007	Turkeys and chickens	A	Serum samples, tracheal tissue	Indirect ELISA, nested RT-PCR, indirect immunofluorescence	[54]
Turkey	2008	Turkeys and chickens	B	Trachea swab and tissue	RT-PCR	[55,56]
India	2014	Chickens	-	Serum samples, choanal cleft swabs	Commercial ELISA, RT-PCR	[57,58]
Greece	2016	Chickens	B	Rhino-pharyngeal and tracheal swab	RT-PCR	[21]
Romania	2016	Chickens	B	Tracheal swab	RT-PCR	[59]
Morocco	2020	Chickens	A and B	Tracheal and turbinate swab, serum samples	RT-PCR, indirect ELISA	[20,60]

**Table 2:** Timeline of aMPV outbreaks in the United States [70,71].

Year	State	Subtype
1997–2007	Colorado, North Dakota, South Dakota, Minnesota, Wisconsin, Indiana	C
	Indiana	C
2014 (limited outbreak)	Wisconsin	C
	Minnesota	C
2023–2024 (limited outbreak)	Indiana	C
December 2023	North Carolina	B
	California	A
January 2024	Virginia	B
	South Carolina	B
7 February 2024	West Virginia	B
	Pennsylvania	B
	Delaware	B
29 February 2024	Georgia	B
	Michigan	B
	Texas	A
	Maryland	B
26 March 2024	Indiana	B
	Arkansas	A
	Illinois	B
9 April 2024	Missouri	A
	Illinois	A
	Ohio	B
	Kentucky	B
	Arkansas	B
17 April 2024	Louisiana	A
	Indiana	A
	Ohio	A
	Wisconsin	A
	Tennessee	B
26 April 2024	Oklahoma	A
	Minnesota	A
	Missouri	B
10 May 2024	Minnesota	B
	Iowa	A
16 May 2024	Alabama	B
7 June 2024	Iowa	B
	South Dakota	A
1 July 2024	South Dakota	B
1 July 2024	North Dakota	A
	Utah	A
2 August 2024	Utah	B
	Michigan	A
29 August 2024	Kansas	A
	Pennsylvania	A
	Kentucky	A
10 March 2025	Tennessee	A
	Alabama	A
	Mississippi	A
	Florida	B
16 May 2025	Georgia	A
	North Carolina	A

**Table 3:** Natural and experimental hosts of aMPV subtypes.

aMPV Subtype	Natural Hosts (Identified)	Experimental Hosts (Tested)
A	Turkey, Chicken, Wild Birds	Turkey, Chicken
B	Turkey, Chicken, Guinea Fowl, Wild Birds	Turkey, Chicken
C	Turkey, Chicken, Ducks (only Eurasian strain), Pheasants, Wild Birds	Turkey and Chicken (Eurasian strain), Mice, Rats
D	Turkey	Chicken

**Table 4:** Cell lines that have been reported for aMPV isolation and propagation.

Cell Line/System	Species/Tissue Origin	Use in aMPV Work	Subtypes	References
Tracheal Organ Culture	Turkey or chicken tracheal rings	Gold standard for primary isolation and propagation	Mainly A, B. No ciliostasis is observed with aMPV-C.	[6,32,138]
Embryonated Eggs	Embryonated turkey or chicken eggs	Primary isolation and propagation	A, B, C	[6,24,141]
Chicken Embryo Related Cells	Chicken/hamster	Primary isolation and propagation	A, B	[144,145]
Chicken Embryo Fibroblasts Cells	Chicken embryo	Primary isolation and propagation	A, B, C	[47,116,141,144]
Chicken Embryo Lung Cells	Chicken embryo	Primary isolation and propagation	A, B	[47,54]
QT-35 Cells	Japanese quail fibrosarcoma	Primary isolation and propagation	A, B, C	[46,146,147]
Turkey Tracheal Cells	Primary cell line from the trachea of 1-day-old turkey poults	Primary isolation and propagation	B	[148]
Vero Cells	African green monkey kidney	Primary isolation and propagation	A, B, C, D	[6,12,94,116,142]
DF-1 Cells	Immortalized chicken fibroblasts	Propagation	C	[149]
Chicken Embryo Liver Cells	Chicken embryo	Propagation	A	[142]
Vero E6 Cells	African green monkey kidney derivative	Propagation	B	[148]
BHK-21 Cells	Baby hamster kidney	Propagation	A, B, C	[144,149,150]
BGM-70 Cells	Baby grivet monkey kidney	Propagation	C	[149,150]
MA-104 Cells	African green monkey kidney	Propagation	C	[149,150]
Turkey Embryo Fibroblasts	Turkey embryo	Propagation	C	[149,150]

**Table 5:** List of imported aMPV vaccines currently authorized for use in the US [71].

Vaccine Name	Manufacturer	Type	Subtype Used	Target Species
Vaxxon^®^ SHS	Vaxxinova, Italy	Modified live	B	Turkeys and chickens
Poulvac^®^ TRT	Zoetis, Spain	Modified live	A	Turkeys and chickens
AVIFFA RTI	Boehringer-Ingelheim, France	Modified live	B	Turkeys and chickens
NEMOVAC^®^	Boehringer-Ingelheim, France	Modified live	B	Chickens
RESPIVAC^®^ aMPV	Hipra, Spain	Modified live	B	Chickens
TUR-3	Boehringer-Ingelheim, France	Killed/inactivated	B	Turkeys and chickens
HIPRAVIAR^®^ TRT	Hipra, Spain	Killed/inactivated	B	Turkeys and chickens

## Data Availability

No new data were created or analyzed in this study. Data sharing is not applicable to this article.

## Abbreviations

The following abbreviations are used in this manuscript:

aMPV	Avian metapneumovirus
TRT	Turkey rhinotracheitis
SHS	Swollen head syndrome
hMPV	Human metapneumovirus
F	Fusion protein
P	Phosphoprotein
M	Matrix protein
N	Nucleoprotein
M2	Second matrix protein
G	Glycoprotein
SH	Small hydrophobic protein
L	Large polymerase protein
NCFAD	National Centre for Foreign Animal Disease
HFS	Hydrosalpinx fluid syndrome
BIC	Bayesian Information Criterion
TMRCA	The most recent common ancestor
BEAST	Bayesian evolutionary analysis by sampling trees
TOC	Tracheal organ culture
OOC	Oviduct organ culture
ISG	Interferon-stimulated gene
IRF3	Interferon regulatory factor 3
CEF	Chicken embryo fibroblast
CEL	Chicken embryo liver cell
BHK	Baby hamster kidney
PI	Post-infection
MDA	Maternally derived antibodies
ISCOM	Immune-stimulating complexes
USAHA	US Animal Health Association
RT-PCR	Reverse transcription polymerase chain reaction
